# Prehospital anaesthesiologists experience with cardiopulmonary resuscitation-induced consciousness in Norway – A national cross-sectional survey

**DOI:** 10.1016/j.resplu.2024.100591

**Published:** 2024-02-29

**Authors:** Jostein Rødseth Brede, Eivinn Årdal Skjærseth, Marius Rehn

**Affiliations:** a Department of Emergency Medicine and Pre-Hospital Services, St. Olav University Hospital, Trondheim, Norway; b Norwegian Air Ambulance Foundation, Department of Research and Development, Oslo, Norway; c Department of Anaesthesiology and Intensive Care Medicine, St. Olav́s University Hospital, Trondheim, Norway; d Air Ambulance Department, Division of Prehospital Services, Oslo University Hospital, Oslo, Norway; e Institute of Clinical Medicine, University of Oslo, Oslo, Norway

**Keywords:** CPR, CPRIC, Resuscitation consciousness, OHCA, Cardiac arrest

## Abstract

**Background:**

During cardiopulmonary resuscitation (CPR) cerebral blood flow may be sufficient to restore some cerebral function, and CPR-induced consciousness (CPRIC) may occur. CPRIC includes signs of life such as gasping, breathing efforts, eye opening, movements of extremities or communication with the rescuers. There is a lack in evidence for prevalence, experience, and possible treatment strategies for CPRIC. This survey aimed to assess prehospital anaesthesiologists experience with CPRIC in Norway.

**Methods:**

A web-based cross-sectional survey. All physicians working at a Norwegian air ambulance, search-and-rescue base or physician-staffed rapid response car were invited to participate.

**Result:**

Out of 177 invited, 115 responded. All were anaesthesiologist, with mean 12.7 (SD 7.2) years of prehospital experience, and 25% had attended more than 200 out-of-hospital cardiac arrests (OHCA). CPRIC was known amongst most physicians prior to the survey and experienced by 91%. Mechanical compression device was used in 79% of cases. The CPRIC were CPR-interfering in 31% of cases. Next-of-kin reported the CPRIC as upsetting in 5% of cases. Medication and/or physical restraint were administered in 75% patients. For patients with CPRIC 50% answered that sedation was needed. If sedation should be provided, 62% answered that this should only be performed by a physician, while 25% answered that both ambulance crew and physicians could provide sedation. Fentanyl, ketamine, and midazolam were suggested as the most appropriate sedation agents.

**Conclusion:**

This nationwide survey indicates that CPRIC during OHCA are well known amongst prehospital anaesthesiologist in Norway. Most patients with CPRIC were treated with chest compression device. Most physicians recommend sedation of patients with CPRIC during resuscitation.

## Background

In out-of-hospital cardiac arrest (OHCA) cardiopulmonary resuscitation (CPR) aims to improve oxygen delivery to the brain and heart, to limit hypoxic cerebral damage and to achieve return of spontaneous circulation (ROSC).^1^ During CPR the blood flow to the brain may be sufficient to restore some cerebral function, without ROSC.^1^, ^2^ Therefore, cardiopulmonary resuscitation-induced consciousness (CPRIC) is emerging as a phenomenon and was recognized in the 2015 guidelines from the European Resuscitation Council.^3^ It includes signs of life such as gasping, breathing efforts, eye opening, movements of extremities or even communication with the rescuers.^2^ CPRIC can further be classified as CPR interfering or CPR non-interfering.^4^ It is not yet included in commonly used cardiac arrest registration templates.

There has been an increased body of evidence since the first systematic review in 2014 which found nine reports with a total of 10 patients.^2^ However, most are case reports, a few prospective studies on patients,^5^, ^6^, ^7^, ^8^ reports on the rescuers’ experience^4^, ^9^, ^10^ and a scoping review,^11^ in addition to a prospective study of in-hospital CPRIC.^12^ This increase in publications may be due to increased prevalence, increased focus on the condition, or both. The reported prevalence of CPRIC was 0.23–0.9%,^11^ but the design of the studies greatly influences the validity of the data. Increased use of mechanical compression devices may also influence the prevalence of CPRIC due to potentially increased cerebral perfusion.

Findings suggest that prehospital healthcare providers commonly experience CPRIC,^4^ with as many as 48–59% of rescuers observing the condition.^11^ Sedation of these patients may be beneficial for the well-being of both patients and rescuers, however there is no consensus on an intra-arrest CPRIC management protocol.^13^

The lack of sound evidence for the prevalence of CPRIC is striking and calls for investigation. Further, there is no data from the Scandinavian countries on the prevalence of CPRIC or the Norwegian experience with this condition. This study therefore aims to establish the prehospital anaesthesiologist experience with CPRIC in Norway through a web-based cross-sectional survey.

## Methods

### Study setting

The Norwegian ground ambulance system is supplemented by a governmentally funded national physician-staffed emergency medical service (EMS) that covers the entire population.^14^ These physicians are consultant anaesthesiologists, which regularly attend OHCAs. The national service consist of seven fixed-wing bases, seven search-and-rescue bases and 11 Norwegian air ambulance bases, and both the search-and-rescue and the air ambulance bases can dispatch in helicopter or a rapid response car.^14^, ^15^

### Study design

This is a cross-sectional and de-identified analysis, with the use of a web-based survey, between 21st September and 9th December 2023. The survey was performed through *Nettskjema* (https://www.nettskjema.no), a web application designed and operated by the University Information Technology Centre at the University of Oslo, designed to meet the privacy requirements in Norway^16^ (Appendix 1 and 2). The survey was developed and designed by the first author (JRB). The survey pilot was tested among the other authors (EAS and MR) for failproof and logical design, as some questions were only available if the participant answered that he/she had experienced CPRIC.

All physicians working at a Norwegian air ambulance, search-and-rescue base or physician-staffed rapid response car was invited to participate in the survey. An electronic mail describing the study design and purpose was sent to all invited participants. Participation in the survey was voluntary and de-identified. Two reminders of the survey were automatically sent to all invited participants after two and four weeks.

In the survey, CPRIC was defined as either breathing, gasping, eye opening, movement of arms or legs and/or communication with the health care provider.

The study is reported in accordance with the strengthening the reporting of observational studies in epidemiology (STROBE) statement guidance.^17^

### Statistical analysis

Normal distribution of data is confirmed by Shapiro-Wilk test. Continuous variables are reported as mean +/- SD. Categorical variables are described as count and/or proportion (%), as appropriate. No statistical analysis is performed. Data is managed with SPSS (IBM Corp. Released 2017. IBM SPSS Statistics for Windows, Version 25.0. Armonk, NY: IBM Corp).

### Ethics

The study was approved by the Norwegian Regional Committee for Medical and Health Research Ethics (reference 650336) and by the data protection officer at the St. Olavs University Hospital (date 14082023). All participants received information in an electronic mail which included invitation to the study. The first page of the survey also acted as a consent form, and thereby provided informed consent to the use of study data. The survey and consent form are available as supplemental material.

## Results

Out of 177 participants invited to the survey, 115 responded (64.9%).

Eighty-four percent (*n* = 97) were male, and most physicians were experienced, with a mean of 12.7 years of prehospital service. Sixty-eight percent (*n* = 79) had attended more than 50 OHCAs and 25% (*n* = 29) more than 200 OHCAs. CPRIC was known amongst most physicians prior to the survey and experienced by 91% (*n* = 105). A chest compression device was used in 79% of CPRIC cases (Table 1). Next-of-kin reported to the physician on scene that the CPRIC was upsetting in five cases.

**Table 1:** Description of the physician’s experience with CPRIC. The questions marked with * were answered only by those that had experienced CPRIC. OHCA indicates out-of-hospital cardiac arrest; CPRIC, cardiopulmonary resuscitation-induced consciousness.

Gender, *n* (%)	
Female	17 (14.8)
Male	97 (84.3)
I do not want to answer	1 (0.9)
Years in prehospital service, mean (SD)	12.67 (7.2)

Numbers of OHCA attended, *n* (%)	
0–10	2 (1.7)
11–20	8 (7)
21–50	26 (22.6)
51–100	25 (21.7)
101–200	25 (21.7)
More than 200	29 (25.2)

Heard of CPRIC prior to survey, *n* (%)	
Yes	88 (76.5)
No	27 (23.5)

Experienced CPRIC, *n* (%)	
Yes	105 (91.3)
No	10 (8.7)

Number of CPRIC cases experienced*, *n* (%)	
0–2	18 (17.1)
3–4	19 (18.1)
5–6	17 (16.2)
7–10	23 (21.9)
11–20	14 (13.3)
More than 20	14 (13.3)

In CPRIC cases, were chest compression device used*, *n* (%)	
Yes	20 (19)
Yes, in some	63 (60)
No	17 (16.2)
I don’t remember	7 (6.7)

**Fig. 1:**
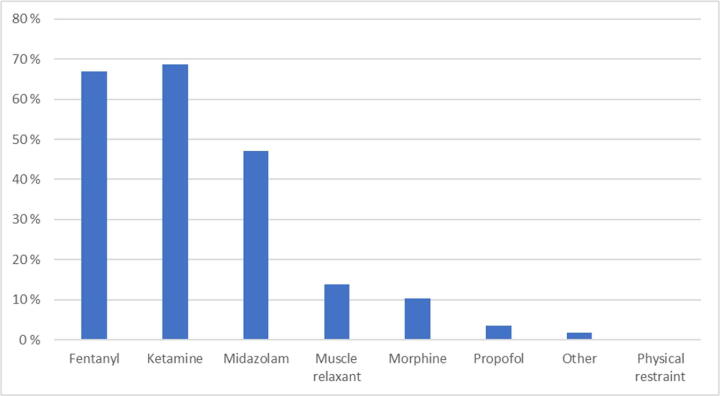
Suggested medication or intervention in case of resuscitation consciousness.

Almost all physicians (95%) answered that sedation of every patient with OHCA is unnecessary. For patients with CPRIC 50% (*n* = 58) answered that sedation is needed, 20% (*n* = 23) answered that no sedation should be provided, and 30% (*n* = 34) was not sure. If sedation should be provided, 62% (*n* = 71) answered that this should only be performed by a physician, 25% (*n* = 29) answered that both ambulance crew and physicians could provide such sedation, while 13% (*n* = 15) was not sure. Most physicians reported fentanyl, ketamine, and midazolam as the most appropriate sedation agent (Fig. 1).

**Fig. 2:**
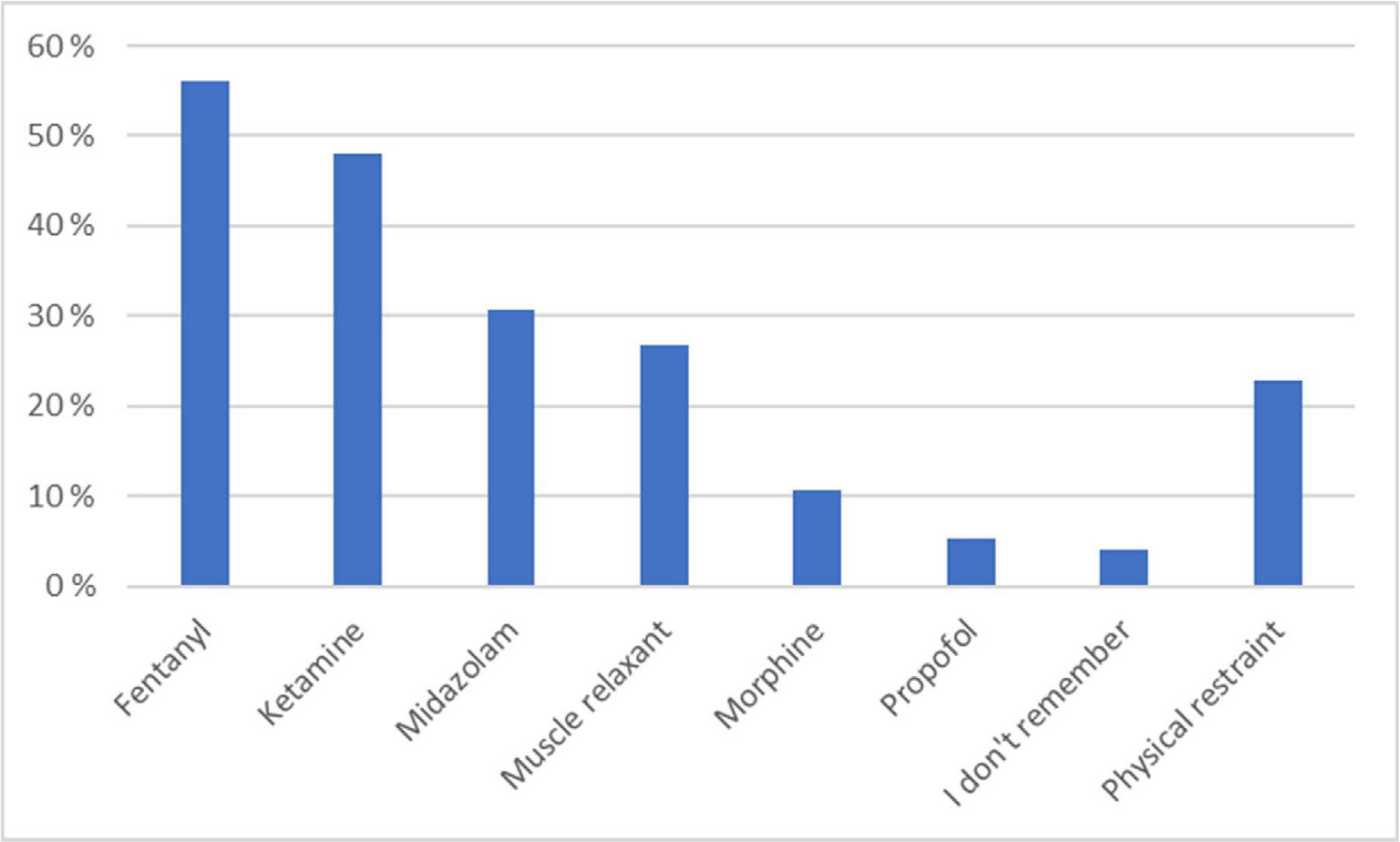
Medication or intervention used due to resuscitation consciousness.

The CPRIC were CPR-interfering in 31% (*n* = 32) of cases. Either medication and/or physical intervention were administered in 75% (*n* = 79) patients (Table 2). Physical restraint included i.e., holding the head still, holding or fastening an extremity, or other physical reduction of movement. The medication provided are shown in Fig. 2.

**Table 2:** Interventions due to CPRIC. These questions were only available for participants that had experienced CPRIC. CPRIC indicates cardiopulmonary resuscitation-induced consciousness; CPR, cardiopulmonary resuscitation.

The CPRIC was CPR-interfering, *n* (%)	
Yes	32 (30.5)
No	66 (62.9)
I don't remember	7 (6.7)

Interventions performed due to CPRIC, *n* (%)	
Physical restraint	4 (3.8)
Medication	55 (52.4)
Physical restraint and medication	20 (19)
No intervention	26 (24.8)

Reason for medication provided	
Analgesia	40 (53.3)
Sedation	47 (62.7)
Amnesia	17 (22.7)
Situational control to enable CPR	40 (53.3)

## Discussion

This nationwide survey found that more than 90% of prehospital anaesthesiologist in Norway had experienced CPRIC in an OHCA setting and that a quarter had experienced CPRIC more than 10 times.

In a study similar to this, Olaussen et al.^4^ reports CPRIC experience amongst health care providers such as nurses, ambulance crew, first responders and physicians. The major difference to our study is the cohort of participants, where our study consists solely of consultant anaesthesiologists. The participants had a mean of 12.7 years of prehospital service, and it is a requirement in Norway to be a consultant anaesthesiologist prior to prehospital EMS engagement. Hence, our participants are highly experienced health-care providers, where approximately half have attended more than 100 OHCA and 25% more than 200 OHCA. It is reasonable to assume that increased experience and more OHCA attended also increase the amount of CPRIC observed, possibly because experience may increase situational awareness and subsequent higher quality in the CPR provided. Further, increased knowledge and a learning curve or ‘*clinical eye*’ for the phenomenon may influence this amount. This may contribute to the finding that more participants had experienced CPRIC (91%) than had heard about it prior to the survey (76%). It is likely that some participants were not aware of CPRIC as a concept or that the phenomenon had a proper name, nevertheless they had experienced patients with signs of life during resuscitation. Hence, in hindsight they could answer that they had experienced CPRIC.

The increased use of mechanical compression facilitates uninterrupted high-quality CPR and may hypothetically increase the risk of CPRIC. The use of mechanical compression devices in Norway varies between health care trust, but the trend is an increased use. The national mean was 28% (range 2–66%) in 2015,^18^ with an increase to 33% (range 3–69%) in 2021.^19^ Some of the studies and case reports on CPRIC are approximately 10 years old or older, hence the reported prevalence of 0.23–0.9% may be too low. Additionally, some patients may be treated with resuscitative endovascular balloon occlusion of the aorta as adjunct to CPR^20^ and may have increased risk of CPRIC due to the reduced vascular distribution volume and potentially increased cerebral perfusion pressure caused by aortic occlusion.

CPRIC may influence not only the patients, but also next-of-kin and rescuers. Parnia et al. reports in two prospective multicentre studies that cardiac arrest survivors may have intense memories from the resuscitation.^6^, ^12^ The authors argue that such experiences may promote emotional damage such as cognitive deficits or post-traumatic stress disorder both for the patients and the rescuers.^6^ In a letter to the editor of Resuscitation, Rice et al argues that ‘*A patient making purposeful movements, even being awake and alert while in cardiac arrest, can have profound emotional and psychological implications on the patient as well as the paramedic providers caring for them’*.^21^ In our data we found that physicians remembered that next-of-kin had reported the CPRIC to be disturbing in five cases. However, this number is likely too low, as this is a cross-sectional analysis of retrospective data as perceived by the physician on scene. Prospective studies should therefore include the impact of CPRIC on both rescuers and next-of-kin, and we propose to add data variables for CPRIC in cardiac arrest reporting templates.

Medication and/or physical intervention were performed in 75% of cases, which indicates that there currently is a common practice to sedate CPRIC cases. This harmonizes with the 50% of participants that answered that sedation should be provided to CPRIC cases and the 30% that answered that they were unsure, with only 20% answering no.

Further, the participant’s suggested medication for CPRIC and the medication that was reported in use, coincide. The majority recommended agents such as fentanyl, ketamine, and midazolam, which together with muscle relaxant were the most used. It is unknown why these agents are preferred, other than that they are the most used medications in our service and are often drawn before arrival on scene. The use may also depend on the physician’s perception of the problem, i.e., the physician believe the patient needs amnesia or the CPRIC is mechanically interfering with the CPR. It may also be due to a consideration on cardiovascular depression that agents such as propofol may provide. If such considerations at all are relevant, given that adrenaline is administered abundantly during resuscitation, is unknown. Muscle relaxants were used in 27% of patients while suggested by only 14% as a recommended medication in a sedation protocol. Further, physical restraint was not suggested as an intervention in CPRIC, however it was reported used in 23% of cases (Fig. 1, Fig. 2). In 53% of the patients, medication was used for situational control to enable CPR. This harmonize with the reported CPR-interfering CPRIC in 31% of patients and indicates that CPRIC often is of such a degree that sedation is needed. We therefore argue that a standardized protocol for sedation of patients with CPRIC is warranted.

Interestingly, 62% of participants responded that sedation should only be performed by a physician. However, the majority of OHCA in Norway is attended by ground ambulance crew only, without involvement of the physician-manned EMS. This is presumably similar to most other services. Hence, to deprive patients that are resuscitated by highly trained ground ambulance crew sedation for CPRIC seems unfair, both for the patient and for the personnel involved in the resuscitation.

### Limitations

The first limitation is the study design, as cross-sectional studies are prone to confounding, selection- and information bias.^22^ One example is recall bias, since this survey did not address the time since the physicians experience with CPRIC. However, a cross-sectional study may easily be performed, and allow a ‘benchmark’ and hypothesis for further Norwegian prospective studies on CPRIC. Secondly, we experienced a low response rate (65%). This need not necessarily affect the validity of the study; however, it may increase the risk for sampling bias. It is possible that some did not respond because they had not heard about, or experienced, CPRIC. This would obviously influence the percentages. Finally, this is retrospective data solely based on the memory of the physicians. However, a strength of this study is that it is a nationwide survey that included all prehospital anaesthesiologist in Norway. The physicians are all very experienced and are likely to remember a phenomenon such as CPRIC, at least if the consciousness was CPR-interrupting.

## Conclusion

This nationwide study indicates that the CPRIC phenomenon during OHCA resuscitation are well known amongst prehospital anaesthesiologist in Norway. Most patients with CPRIC were treated with chest compression device. Most physicians recommend sedation of patients with CPRIC during resuscitation.

## Funding

The funding for open access is granted by Department of Emergency Medicine and Pre-Hospital Services, St. Olav University Hospital, Trondheim, Norway. The authors or collaborators received no external funding.

## Availability of data and materials

The dataset used may be made available from the corresponding author upon reasonable request.

## CRediT authorship contribution statement

**Jostein Rødseth Brede:** Writing – original draft, Project administration, Methodology, Investigation, Funding acquisition, Formal analysis, Data curation, Conceptualization. **Eivinn Årdal Skjærseth:** Writing – review & editing, Project administration, Methodology. **Marius Rehn:** Writing – review & editing, Methodology.

## Declaration of competing interest

The authors declare the following financial interests/personal relationships which may be considered as potential competing interests: The authors declare that they have no competing interest. JRB are partly employed at the Norwegian Air Ambulance Foundation for research purposes and MR has received funding from the Norwegian Air Ambulance Foundation for research purposes.
