# Supplementary material for: Prehospital anaesthesiologists experience with cardiopulmonary resuscitation-induced consciousness in Norway – A national cross-sectional survey
Source: Resusc Plus. 2024 Feb 29;18:100591. doi: 10.1016/j.resplu.2024.100591 (PMC10910154; doi:10.1016/j.resplu.2024.100591)
Supplement: Supplementary data 1 [file mmc1.pdf]

CPR-induced consciousness in out-of-hospital cardiac arrest

Background

During cardiopulmonary resuscitation, some patients may show signs of life, so called CPR-induced consciousness (CPR-IC).

This consciousness include:
Breathing
Gasping
Eye opening
Movement of arms/legs AND/OR
Communication with the health care providers.

This survey aims to assess the pre-hospital anaesthesiologists experience with CPR-IC in Norway. Anaesthesiologists working at the air ambulance/rapid response car/rescue helicopter bases are invited to participate.

The survey is approved by the Regional Committees for Medical and Health Research Ethics. The survey is de-identified, no identifiable data will be stored, and participation is voluntary. Questions such as work base, age or gender are not connected, so it is impossible for us to know the origin of the answers.

The survey requires approximately 5 minutes.

By answering «Yes» on the question below, you accept that the information provided is used for scientific purposes.
If you do not want to participate, you can close this window.

Do you wish to participate in this survey?

Yes

General information about you and your experience.

Age

20-29
30-39
40-49
50-59
60 or older

Gender

Female
Male
I do not want to answer

How many years have you worked as a pre-hospital physician?

Which base do you primarily work at?

If more bases, select the one where you have the most shifts.
LA Kirkenes
LA Tromsø
LA Harstad
LA Brønnøysund
LA Rosten
LA Ålesund
LA Førde
LA Bergen
LA Stavanger
LA Arendal
LA Lørenskog
LA Ål
LA Dombås
Bil Oslo (119)
Bil Drammen
330 Rygge
330 Banak
330 Ørlandet
330 Sola
330 Bodø
CHC Florø
CHC Tromsø
SAR Svalbard
I do not want to answer

How many out-of-hospital cardiac arrests have you experienced as a physician?

An approximately estimate.
0-10
11-20
21-50
51-100
101-200
More than 200

Had you heard of CPR-IC prior to this survey?

Yes

No

Have you experienced a patient with CPR-IC?

CPR-IC includes attempted breathing, gasping, eye opening, movement of arms/legs AND/OR communication with the health care providers.
Yes
No

If Yes, how many cases of CPR-IC have you experienced?

This will only be displayed if «Yes» is selected.
Approximately estimate
0-2
3-4
5-6
7-10
11-20
More than 20

About treatment of CPR-IC

Do you think all patients in cardiac arrest should be given sedation during CPR?

Yes
No
I don’t know

Should patients with CPR-IC receive sedation?

Yes
No
I don’t know

Who should provide such sedation?

Both ambulance crew and physicians
Physicians only
I don’t know

What medications do you think should primarily be used?

Multiple answers are possible.
Propofol
Ketamine
Midazolam
Fentanyl
Morphine
Muscle relaxant
External force, holding etc.
Other

Which other medications?

This will only be displayed if «Other» is selected.

Here we ask about your thoughts on interventions and treatment of CPR-IC
We would like to know more about the case/cases where you experienced CPR-IC.
In the case/cases with CPR-IC, where mechanical chest compression device used?

This will only be displayed if «Yes» is selected in the question «Have you experienced a patient with CPR-IC?»

Yes
Yes, in some
No
I do not remember

Was CPR influenced by the CPR-IC?

This will only be displayed if «Yes» is selected in the question «Have you experienced a patient with CPR-IC?»

Yes
No
I do not remember

Was any intervention performed due to the CPR-IC?

This will only be displayed if «Yes» is selected in the question «Have you experienced a patient with CPR-IC?»

If you have experienced more cases, think on the one/ones you performed interventions.
Physical intervention (e.g., holding or securing an extremity, hold the head or similar)
Medication provided
Physical intervention AND medication provided
No intervention

If you provided medication, which medication were used?

This will only be displayed if «Medication provided or Physical intervention AND medication provided» is selected in the question «Was any intervention performed due to the CPR-IC?»

Propofol
Ketamine
Midazolam
Fentanyl
Morphine
Muscle relaxant
I do not remember

What was the reason you gave medication?

This will only be displayed if «Medication provided or Physical intervention AND medication provided» is selected in the question «Was any intervention performed due to the CPR-IC?»

Analgesia
Sedation

Amnesia
Situation control to provide CPR
I do not remember

Did the next-of-kin feel state the CPR-IC was disturbing for them?

This will only be displayed if «Yes» is selected in the question «Have you experienced a patient with CPR-IC?»

Yes
No
I do not remember
