# Supplementary material for: Prehospital anaesthesiologists experience with cardiopulmonary resuscitation-induced consciousness in Norway – A national cross-sectional survey
Source: Resusc Plus. 2024 Feb 29;18:100591. doi: 10.1016/j.resplu.2024.100591 (PMC10910154; doi:10.1016/j.resplu.2024.100591)
Supplement: Supplementary data 2 [file mmc2.pdf]

Nettskjema

HLR-indusert bevissthet ved prehospital hjertestans

Bakgrunn

Ved hjerte-lunge-redning kan enkelte pasienter vise tegn til bevissthet, såkalt HLR-indusert bevissthet eller CPR-induced consciousness (CPR-IC).

Bevisstheten inkluderer:
pusteforsøk
gjesping
øyeåpning
bevegelse av armer/bein OG/ELLER
kommunikasjon med helsepersonellet.

Denne spørreundersøkelsen søker å kartlegge erfaringen norske leger i luftambulansetjenesten har med CPR-IC. Anestesileger som arbeider ved luftambulansebasene/bilambulansene/redningshelikopterbasene blir invitert til å delta.

Undersøkelsen er REK-godkjent. Den er anonym, det vil ikke bli lagret identifiserbare data fra deg og det er frivillig å delta. Spørsmål som f.eks basetilhørighet, alder og kjønn er ikke koblet til hverandre, så det er umulig for oss å vite hvem som har svart hva fra hvilken base.

Det tar ca 5 minutter å besvare undersøkelsen.

Ved å svare "Ja" på spørsmålet under innebærer det at du godtar du at informasjonen du oppgir kan brukes til forskning.
Dersom du ikke ønsker å delta på undersøkelsen kan du lukke dette vinduet.

Ønsker du å delta på spørreundersøkelsen?

Ja

Generell informasjon om deg og din erfaring.

Alder

20-29
30-39
40-49
50-59
60 eller eldre

Kjønn

Kvinne
Mann
Jeg ønsker ikke oppgi dette

Hvor mange år har du jobbet som lege prehospitalt?

Nettskjema

Hvilken base jobber du primært på?

Dersom flere baser, velg den der du går flest vakter.

- LA Kirkenes
- LA Tromsø
- LA Harstad
- LA Brønnøysund
- LA Rosten
- LA Ålesund
- LA Førde
- LA Bergen
- LA Stavanger
- LA Arendal
- LA Lørenskog
- LA Ål
- LA Dombås
- Bil Oslo (119)
- Bil Drammen
- 330 Rygge
- 330 Banak
- 330 Ørlandet
- 330 Sola
- 330 Bodø
- CHC Florø
- CHC Tromsø
- SAR Svalbard
- Ønsker ikke å oppgi dette

Hvor mange prehospitale hjertestanser har du behandlet som lege?

Et omtrentlig anslag

- 0-10
- 11-20
- 21-50
- 51-100
- 101-200
- Mer enn 200

Hadde du hørt om CPR-IC før denne spørreundersøkelsen?

CPR-IC inkluderer pusteforsøk, gjesping, øyeåpning, bevegelse av armer/bein OG/ELLER kommunikasjon med helsepersonellet

- Ja
- Nei

Har du noen gang opplevd en pasient med CPR-IC?

Nettskjema

Ja

Nei

Dersom ja, hvor mange tilfeller av CPR-IC har du opplevd?

Dette elementet vises kun dersom alternativet «Ja» er valgt i spørsmålet «Har du noen gang opplevd en pasient med CPR-IC?»

Omtrentlig anslag

0-2

3-4

5-6

7-10

11-20

Mer enn 20

Om behandling av CPR-IC

Her spør vi om dine tanker rundt eventuelle tiltak og behandling av CPR-IC

Syntes du alle pasienter med hjertestans bør få sedasjon under pågående HLR?

Ja

Nei

Jeg vet ikke

Bør pasienter som viser tegn til CPR-IC få sedasjon?

Ja

Nei

Jeg vet ikke

Hvem bør kunne gi slik sedasjon?

Både ambulansepersonell og leger

Kun leger

Jeg vet ikke

Hvilke medikamenter mener du primært bør benyttes?

Flere valg er mulig.

Propofol

Ketamin

Midazolam

Fentanyl

Morfin

Muskelrelaksantia

Mekanisk tvang, holding etc

Andre

Hvilke andre medikamenter?

Dette elementet vises kun dersom alternativet «Andre» er valgt i spørsmålet «Hvilke medikamenter mener du primært bør benyttes? »

Her vil vi vite mer om den gangen/de gangene du opplevde CPR-IC

Ved tilfellet/tilfellene av CPR-IC, ble det brukt mekanisk kompresjonsmaskin?
Dette elementet vises kun dersom alternativet «Ja» er valgt i spørsmålet «Har du noen gang opplevd en pasient med CPR-IC?»

- Ja
- Ja, ved noen av de
- Nei
- Jeg husker ikke

Ble HLR forstyrret av CPR-IC?
Dette elementet vises kun dersom alternativet «Ja» er valgt i spørsmålet «Har du noen gang opplevd en pasient med CPR-IC?»

- Ja
- Nei
- Jeg husker ikke

Ble det utført tiltak som følge av CPR-IC?
Dette elementet vises kun dersom alternativet «Ja» er valgt i spørsmålet «Har du noen gang opplevd en pasient med CPR-IC?»

Dersom du har opplevd flere tilfeller, tenk på det tilfellet/de tilfellene der du eventuelt utførte tiltak.

- Fysisk inngripen (f.eks holde eller binde fast en ekstremitet, holde hodet i ro e.l.)
- Medikamenter gitt
- Fysisk inngripen OG medikamenter gitt
- Ingen tiltak ble gjort

Dersom du ga medikamenter, hvilke medikamenter ble gitt?
Dette elementet vises kun dersom alternativet «Medikamenter gitt eller Fysisk inngripen OG medikamenter gitt» er valgt i spørsmålet «Ble det utført tiltak som følge av CPR-IC? »

- Propofol
- Ketamin
- Midazolam
- Fentanyl
- Morfin
- Muskelrelaksantia
- Jeg husker ikke

Hva var årsaken til at du ga medikamentene?
Dette elementet vises kun dersom alternativet «Medikamenter gitt eller Fysisk inngripen OG medikamenter gitt» er valgt i spørsmålet «Ble det utført tiltak som følge av CPR-IC? »

- Analgesi
- Sedasjon
- Amnesi
- Situasjonskontroll for å få utført HLR
- Jeg husker ikke

Ga pårørende uttrykk for at de følte at pasientens CPR-IC var problematisk?
Dette elementet vises kun dersom alternativet «Ja» er valgt i spørsmålet «Har du noen gang opplevd en pasient med CPR-IC?»

- Ja
- Nei
- Jeg husker ikke
